# Non‐Toxic Virucidal Macromolecules Show High Efficacy Against Influenza Virus Ex Vivo and In Vivo

**DOI:** 10.1002/advs.202001012

**Published:** 2020-12-14

**Authors:** Ozgun Kocabiyik, Valeria Cagno, Paulo Jacob Silva, Yong Zhu, Laura Sedano, Yoshita Bhide, Joelle Mettier, Chiara Medaglia, Bruno Da Costa, Samuel Constant, Song Huang, Laurent Kaiser, Wouter L. J. Hinrichs, Anke Huckriede, Ronan Le Goffic, Caroline Tapparel, Francesco Stellacci

**Affiliations:** ^1^ Insitute of Materials École Polytechnique Fédérale de Lausanne Station 12 Lausanne 1015 Switzerland; ^2^ Department of Microbiology and Molecular Medicine University of Geneva Rue Michel Servet 1 Geneva 1205 Switzerland; ^3^ Virologie et Immunologie Moleculaire Institut National Recherche Agronomique Université Paris‐Saclay Jouy en Josas 78350 France; ^4^ Department of Pharmaceutical Technology and Biopharmacy University of Groningen Groningen 9713GZ The Netherlands; ^5^ University Medical Center Groningen Department of Medical Microbiology and Infection Prevention (internal postcode EB88) University of Groningen Hanzeplein 1 Groningen 9713GZ The Netherlands; ^6^ Epithelix Sas Chemin des Aulx 18 Geneva 1228 Switzerland; ^7^ Hopital Universitaire de Genève Rue Gabrielle Perret Gentil 4 Geneva 1205 Switzerland; ^8^ Bioengineering Institute Ecole Polytechnique Fédérale de Lausanne Station 12 Lausanne 1015 Switzerland

**Keywords:** 3’SLN, 6’SLN, antivirals, Influenza, virucidal

## Abstract

Influenza is one of the most widespread viral infections worldwide and represents a major public health problem. The risk that one of the next pandemics is caused by an influenza strain is high. It is important to develop broad‐spectrum influenza antivirals to be ready for any possible vaccine shortcomings. Anti‐influenza drugs are available but they are far from ideal. Arguably, an ideal antiviral should target conserved viral domains and be virucidal, that is, irreversibly inhibit viral infectivity. Here, a new class of broad‐spectrum anti‐influenza macromolecules is described that meets these criteria and display exceedingly low toxicity. These compounds are based on a cyclodextrin core modified on its primary face with long hydrophobic linkers terminated either in 6'sialyl‐*N*‐acetyllactosamine (6’SLN) or in 3’SLN. SLN enables nanomolar inhibition of the viruses while the hydrophobic linkers confer irreversibility to the inhibition. The combination of these two properties allows for efficacy in vitro against several human or avian influenza strains, as well as against a 2009 pandemic influenza strain ex vivo. Importantly, it is shown that, in mice, one of the compounds provides therapeutic efficacy when administered 24 h post‐infection allowing 90% survival as opposed to no survival for the placebo and oseltamivir.

## Graphical abstract

Non‐toxic sialylated cyclodextrin derivatives inhibit influenza virus with a virucidal mechanism. Modified cylodextrins are effective against several human and avian strains in vitro. More importantly, one of the compounds shows therapeutic efficacy against an H1N1 pandemic strain when administered 24 h post‐infection in human‐derived respiratory tissues and in vivo.

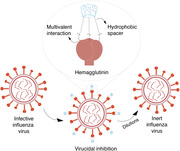

Influenza viruses are among the most infective viruses.^[^ ^1^, ^2^ ^]^ Every year, different influenza strains infect a large fraction of both the animal and human population^[^ ^3^ ^]^ endangering infants, the elderly, and immunocompromised people, which are at high risk of hospitalization and death, due to influenza‐related complications.^[^ ^4^, ^5^, ^6^, ^7^, ^8^ ^]^ As a result, seasonal influenza has yearly a remarkable socio‐economic impact. Respiratory diseases can cost a significant fraction of the total health expenditures in developed and mainly in developing countries.^[^ ^9^, ^10^ ^]^ Because influenza mutates so rapidly, the development of a lifelong vaccine is still a major challenge.^[^ ^11^, ^12^, ^13^ ^]^ Vaccine development would pose even higher challenges when we focus on the occasional pandemics instead of yearly outbreaks. In such a case, the development time of a new vaccine would represent a serious risk. Furthermore, even in the presence of a vaccine, reaching reasonable vaccination coverage is far from a foregone conclusion. As a consequence, the risk of a new pandemic, such as the Spanish‐flu, is still present and recognized as one of the top threats to global health.^[^ ^14^, ^15^, ^16^ ^]^

Naturally, the second line of defense after vaccines, are antiviral drugs. A number of anti‐influenza drugs are currently approved: neuraminidase inhibitors such as zanamivir and oseltamivir, ion channel inhibitors such as amantadine, fusion inhibitors such as umifenovir (only in Russia and China) and polymerase inhibitor such as baloxavir marboxil, which was recently approved in the US and Japan. Yet, it is recognized that the efficacy of current drugs is far from ideal. Concerns about these drugs range from significant side effects to the appearance of drug‐resistant viruses after a short period of use.^[^ ^17^ ^]^ Given the importance of this issue, a number of other antivirals are in clinical trials.^[^ ^18^, ^19^, ^20^, ^21^, ^22^, ^23^, ^24^, ^25^ ^]^ The majority of these drugs are monoclonal antibodies^[^ ^26^, ^27^ ^]^ that inhibit the fusion of the virus to the host cell. They are promising, but it is likely that they will be costly due to their manufacturing processes. Furthermore, monoclonal antibodies are expected to be good prophylactic drugs, but their efficacy in therapeutic administration (i.e., post infection) is a matter of intense research.

An ideal anti‐influenza drug should be broad‐spectrum, by targeting a highly conserved part of the virus and, in order to avoid loss of efficacy due to the dilution in body fluids, has an irreversible effect, that is, be virucidal. Obviously, this drug needs to be truly non‐toxic. There are quite a few research lines on the development of molecules that target conserved parts of the virus.^[^ ^28^, ^29^, ^30^, ^31^, ^32^, ^33^, ^34^, ^35^, ^36^, ^37^, ^38^, ^39^, ^40^, ^41^ ^]^ Peptide‐based compounds have shown convincing in‐vivo results in therapeutic settings.^[^ ^41^ ^]^ The situation is different for compounds that are fully chemical (and hence inexpensive to manufacture). These compounds employ elegant multivalent strategy and reach very low inhibitory concentrations;^[^ ^33^, ^34^, ^35^, ^36^, ^37^, ^38^ ^]^ but, to the best of our knowledge, these compounds are all reversible in their action and hence could face hurdles when translating into drugs. Indeed, to date, no multivalent compound with broad‐spectrum anti‐influenza efficacy has shown convincing post‐infection results in vivo of the type that we present here.

The search for virucidal (i.e., irreversible) drugs with limited toxicity has been very challenging. Polymers bearing hydrophobic groups have previously been reported to show virucidal activity or enhanced antiviral activity.^[^ ^42^, ^43^ ^]^ We have also shown that gold nanoparticles^[^ ^44^ ^]^ and *β*‐cyclodextrins^[^ ^45^ ^]^ (β‐CDs) modified with 11‐undecane sulfonic acid display a virucidal mechanism against a wide range of heparan sulfate proteoglycans (HSPGs) binding viruses, with no cellular toxicity. These compounds were capable of exerting forces that ultimately deform the virus particle. The chemical structure of the ligand was shown to be essential in order to achieve such irreversible inhibition.

Here, we adopted a similar strategy in order to target human influenza viruses and avian influenza viruses. 6’ sialyl‐N‐acetyllactosamine (6’SLN) and 3’ sialyl‐*N*‐acetyllactosamine (3’SLN), that specifically binds to hemagglutinin (HA) trimers of influenza strains,^[^ ^46^ ^]^ were grafted onto the primary face of *β*‐CDs through a series of different linkers. The structure of all modified cyclodextrins discussed in this work is shown in **Figure** **1**. Of note, since each HA trimer has three sialic acid binding pockets, we aimed to modify the *β*‐CDs with three trisaccharides. Therefore, the cyclodextrins are not fully modified but they all bear, on average, a comparable number of trisaccharides (Figures S1 and S2, Supporting Information), determined using ^1^H Nuclear Magnetic Resonance Spectroscopy (NMR). In vitro dose–response assays against influenza A/Netherlands/602/2009 (H1N1) strain (A/NL/09), were conducted to compare the inhibitory activity of these molecules (Figure 1 and Figure S3, Supporting Information). The infection was quantified with immunocytochemical assays at 24 h post‐infection (hpi). *β*‐CDs bearing a sufficiently long, hydrophobic linker and 6’SLN end‐group (**C6‐6’**, **C11‐6’**, and **C14‐6’**) showed strong inhibitory activity against infection with influenza A/NL/09, having EC_50_ values in the nanomolar range. On the other hand, the *β*‐CD with a shorter linker, **C1‐6’**, poorly protects from the infection. Introducing a sufficiently long linker clearly enhanced the end‐group flexibility; hence the inhibitory concentrations decreased. The EC_50_ was comparable (yet slightly higher) when the hydrophobic linker was replaced with a hydrophilic poly(ethylene glycol) (8) (PEG8) linker (**P8‐6’**).

**Figure 1:**
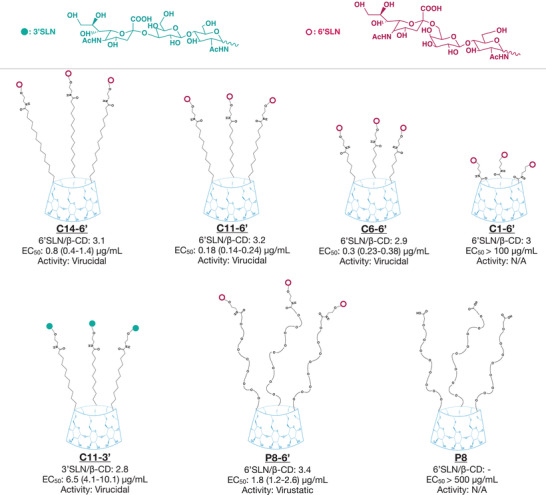
Summary of the modified cyclodextrins. The average number of 6’SLN or 3’SLN per *β*‐CD was calculated by ^1^H NMR. The representative chemical structures of modified cyclodextrins were constructed based on NMR results. EC_50_ represents the half‐effective concentrations on MDCK cells at 24 hpi against A/NL/09 with the respective 95% confidence interval (CI) (Figure S3, Supporting Information). N/A: not assessable.

In order to demonstrate viral inhibition is due to trisaccharide group but not due to *β*‐CD or the linker bearing carboxylic acid end group, we also tested *β*‐CD solely modified with PEG8 linker **(P8)**. P8 did not inhibit influenza A/NL/09, even at very high material concentrations.

**C11‐6’**, the molecule that showed the best inhibitory activity against A/NL/09, displayed strong antiviral activity against human influenza strains from both the A (H1N1 and H3N2) and the B type (**Table** **1** and Figure S4, Supporting Information). Importantly, it inhibited very recent A (H1N1) and B clinical strains (from the 2017/2018 influenza season), isolated from patients in the University Hospital of Geneva and passaged only once in Madin‐Darby Canine kidney (MDCK) cells. **C11‐6’** did not show any antiviral activity against herpes simplex virus 2 (HSV‐2), an HSPG‐binding virus, indicating the specificity of the compound for sialic acid dependent viruses.

**Table 1:** Inhibitory activity of C11‐6’ and C11‐3’ against different influenza strains

	Compound	CC_50_ [µg mL^−1^]	EC_50_ [µg mL^−1^]	EC_50_ ^a)^ [nm]
A/Netherlands/602/2009 (H1N1)	C11‐6’	>100	0.18 (0.14 – 0.24)	42
	C11‐3’	>100	6.5 (4.1‐10.1)	>1000
A/Switzerland/8337/2018‐MDCK1 (H1N1)	C11‐6’	>100	0.5 (0.4 – 0.67)	125
A/Singapore/37/2004 (H3N2)	C11‐6’	>100	0.23 (0.16 – 0.34)	56.5
B/Wisconsin/01/2010^b)^	C11‐6’	>100	2.2 (1.49 – 3.42)	500
B/Switzerland/3849/2018‐MDCK1^b)^	C11‐6’	>100	20 (10.5 – 28.7)	>1000
A/turkey/Turkey/2005 (H5N1)	C11‐3’	>100	4.1 (2.55‐6.7)	931
	C11‐6	>100	N/A	N/A
A/turkey/Italy/1999 (H7N1)	C11‐3’	>100	8.8 (3.2‐26)	>1000
HSV‐2 (Control)	C11‐6’	>100	N/A	N/A

^a)^ Molar concentrations were determined based on the modified cyclodextrins

^b)^ B Yamagata lineage

CC_50_: Half‐cytotoxic concentration. EC_50_: Half‐effective concentration with 95% CI in brackets. N/A: not assessable

6’SLN is known to be specific to human influenza strains, whereas 3’SLN is preferred by avian influenza strains as a primary attachment point.^[^ ^47^ ^]^ To prove the generality of our approach, especially against influenza strains that are known to have the ability to cross the species barrier, we synthesized and tested **C11‐3’** (Figure 1) against avian influenza strains. We show that **C11‐3’** inhibits both H5N1 and H7N1 avian strains, at 4.1 and 8.8 µg mL^−1^ concentrations respectively (see Table 1). De facto, these results confirm the antiviral strategy adopted against human strains. We additionally tested whether **C11‐3’** could inhibit the human strain A/NL/09 and whether **C11‐6’** would also be active against the avian strain, H5N1. **C11‐3’** displayed good inhibitory activity against A/NL/09 (Figure 1 and Table 1), whereas **C11‐6’** did not show any activity against H5N1 (Table 1 and Figure S5, Supporting Information). These results are in line with previous literature comparing the binding affinities of avian and human strains to the different types of sialic acids.^[^ ^47^, ^48^ ^]^ Avian influenza strains (particularly H5N1 strains) preferentially bind to alpha ‐2,3 linked sialic acid, which has a thin and straight trans conformation. On the other hand, the wider sialic acid binding site of human strains can accommodate both the bulky cis conformation of alpha ‐2,6 linked sialic acid and the narrower ‐2,3 linked sialic acid.^[^ ^47^, ^48^ ^]^

The synthesis of similar compounds sharing the *β*‐cyclodextrin core and the 6’SLN moiety but different linkers allowed us to highlight a structural feature conferring irreversible inhibitory activity, that is, virucidal action (**Figure** **2** and Figure S6, Supporting Information). This feature differs from the one described in our previous work where only the length of the linkers was compared.^[^ ^44^ ^]^ Here, we hypothesized that one of the key components of an irreversible viral inhibition is that the binding moiety (here 6’SLN) is borne by a sufficiently long hydrophobic linker. A hydrophilic linker such as PEG should not be capable of generating forces that permanently inactivate the virus. To test this hypothesis, we compared **C11‐6’** and **P8‐6’**. These two compounds differ solely in the hydrophobicity of the linker and show comparable inhibitory activity against A/NL/09, as shown on the left in Figure 2a,b. Virucidal assays were conducted as previously described^[^ ^44^ ^]^ to compare the mechanism of inhibition of these compounds, that is, virucidal (irreversible) or virustatic (reversible). Briefly, amounts of the compounds that provide complete protection (10 µg of **C11‐6’** and 50 µg of **P8‐6’**) were incubated with the virus for 1 h. Serial dilutions of the inocula were conducted followed by an evaluation of the infectivity. In the case of **P8‐6’**, the right graph in Figure 2a shows that while at the initial concentration complete protection was present, upon dilution the difference with the infectivity of the control sample (virus alone) was lost, that is, the inhibitory effect was found to be reversible (virustatic). In the case of **C11‐6’** the right graph in Figure 2b shows that complete protection was kept upon dilution and the graphs in Figure 2c show that this property was conserved against a number of different strains.

**Figure 2:**
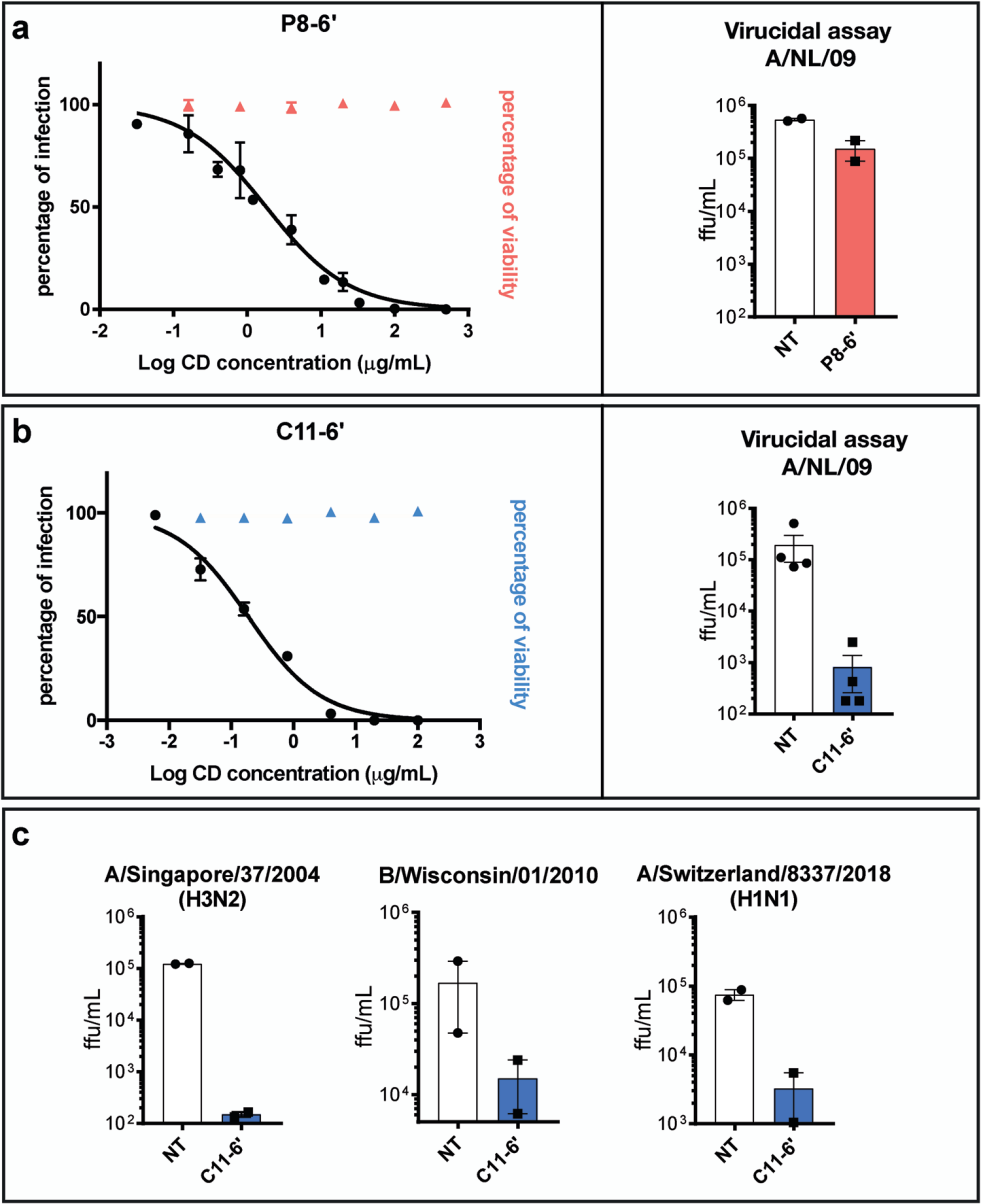
Antiviral activity comparison of C11‐6’ and P8‐6’ in vitro. a,b) These panels show on the graphs on the left the inhibitory activity of each compound against A/NL/09, superimposed with the results of the cell viability assays. Both of the compounds inhibit the virus in the dose–response assay. In the virucidal assays on the right, **C11‐6’** reduced the virus titer by 1000 times, whereas the infection was fully recovered in the case of **P8‐6’**. Hence **C11‐6’** has an irreversible inhibitory effect on the virus while the effect of **P8‐6’** is reversible. c) Virucidal activity of **C11‐6’** against other influenza strains was further investigated confirming its irreversible activity independently of the strain. Note that in the figure's axes **ffu** stands for focus forming units and **NT** for non‐treated. In Figure 2c the following viral strains were tested: A/Singapore/37/2004 (H3N2), B/Wisconsin/01/2010 and A/Switzerland/8337/2018‐MDCK1 (H1N1) Figure 2c. Results are mean and SEM of 2 independent experiments performed in duplicate.

To further compare **C11‐6’** and **P8‐6’**, we performed ex vivo experiments in MucilAir, a 3D model of human airway reconstituted epithelia. These air‐liquid interface cultures perfectly mimic both the pseudostratified architecture (basal, ciliated, and goblet cells) and the barrier defense mechanism (i.e., the mucociliary clearance and epithelial cell immunity) of the human upper respiratory epithelium, the main site of influenza virus replication in humans. Ex vivo experiments were conducted with clinical H1N1 pandemic 09 strain (A/Switzerland/3076/16) that has not been passaged in cells to exclude any adaptation bias. **C11‐6’** or **P8‐6’** (50 µg/tissue) and the virus (10^4^ RNA copies/tissue) were first added simultaneously on the apical surface of the tissues, without prior incubation. After four hours, the inocula were removed, the tissues were washed and the progress of the infection was monitored on daily basis with quantitative polymerase chain reaction (qPCR) performed on viruses isolated from the apical washes of the tissue, without any re‐addition of the molecule. **C11‐6’** completely prevented virus replication throughout the entire course of the experiment, while **P8‐6’** slightly reduced viral replication the first two days post‐infection (dpi) but not thereafter (**Figure** **3**a).

**Figure 3:**
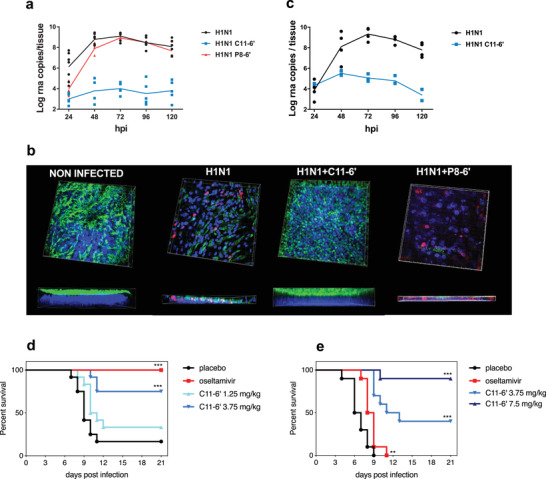
Ex vivo (a to c) and in vivo (d to e) inhibitory activity of C11‐6’. a) Ex vivo, C11‐6’ provided full protection against clinical H1N1 pandemic 09 strain in co‐treatment condition, whereas P8‐6’ only provided minor protection at the beginning of the infection. b) Immunofluorescence at 7 days post‐infection (co‐treatment condition) confirms the protection provided by C11‐6’. Red: monoclonal antibody influenza A, blue: DAPI, green: *β*‐IV‐tubulin (marker of ciliated cells). The thickness of each tissue is shown at the bottom of the corresponding image. c) C11‐6’ also showed high efficacy in post‐treatment condition. Results of (a) and (c) are mean and SEM of 2 to 4 independent experiments with intra‐experimental duplicates. Images of (b) are representative of 10 images taken for each condition. In vivo, mice (12/group) were intra‐nasally treated with PBS or C11‐6’ or by oral gavage with oseltamivir and infected with A/California/09. d) Subsequent treatments were administered daily for the following 5 days, image shows the survival curve. e) Mice were infected and treated with C11‐6’ or oseltamivir at 24 hpi and daily for the 4 following days, images show the survival curves ***p<0.001, **p<0.01

Moreover, in the tissues treated with **C11‐6’**, the inhibition of viral replication was also reflected by the absence of infected cells and the undisturbed morphology of the treated tissues, strikingly different from the untreated or **P8‐6’**‐treated tissues (Figure 3b). Immunofluorescence images and the lack of lactate dehydrogenase release in the apical washes demonstrated that the ciliated cell layer as well as the physiological cilia beating and tissue integrity were preserved (Figure 3b and Figure S7, Supporting Information). In stark contrast, the untreated tissue or the **P8‐6’**‐treated controls, presented reduced thickness due to alteration of the ciliated cell layer, and the presence of infected cells (Figure 3b). To exclude that the residual viral level detected by qPCR in the treated tissues was related to active replication, we kept the tissues in culture for 23 days but never observed an increase in viral titer over time, while the untreated tissues were persistently shedding virus as previously reported^[^ ^49^ ^]^ (Figure S8, Supporting Information). Importantly, ex vivo experiments were conducted also in more stringent post‐treatment conditions in which **C11‐6’** (30 µg/tissue) was administrated every 24 h and for 4 days, starting at 1 dpi to mimic a therapeutic administration. Also in these conditions, the compound showed a remarkable inhibitory activity, proving its potential as a therapeutic agent (Figure 3c). In the same ex vivo model, we also evaluated the biocompatibility of high doses of **C11‐6’**, administered daily. **C11‐6’** did neither show any cytotoxic nor pro‐inflammatory activity in the above described conditions (Figure S9, Supporting Information).

Lastly, in vivo experiments were conducted with BALB/c mice administered first with **C11‐6’** (1.25 or 3.75 mg kg^−1^) and immediately after with A/California/09 via the intranasal route and subsequently treated daily for 5 days. The weights of the mice were measured daily in order to estimate the impact of **C11‐6’** administration on the infected animal's physiological condition (weight loss variation is shown in Figure S11, Supporting Information). A significant increase in survival was observed in presence of **C11‐6’** 3.75 mg kg^−1^ (9/12 mice) and the oseltamivir 30 mg kg^−1^/day (10/10 mice) if compared to placebo control (3/12 mice). Additional experiments were carried out with the administration of 1.25 mg kg^−1^ of **C11‐6’** at the time of infection by measuring the viral load in the lungs and the viral titer in the broncho‐alveolar lavages at 2dpi (Figure S11, Supporting Information). The significative reduction in presence of 1.25 mg kg^−1^ of **C11‐6’** demonstrates that a single administration before the infection is able to significantly decrease the infectious titer of the virus. Subsequently, we performed a post‐treatment experiment, in which mice were treated at 8 (Figure S12, Supporting Information) or 24 hpi (Figure 3d) with **C11‐6’** (3.75 mg kg^−1^ or 7.5 mg kg^−1^) or oseltamivir (30 mg kg^−1^/day). In presence of **C11‐6’** with the start of treatment at 24hpi 9 out of 10 mice survived to the viral challenge in the 7.5 mg kg^−1^ group and 4 in the 3.75 mg kg^−1^ group, in contrast with oseltamivir in which group 0 out of 10 survived. Collectively, these results suggest a potent prophylactic and therapeutic capacity of the **C11‐6’** compound in vivo.

In summary, we present here a new design rule to produce effective, non‐toxic, and virucidal compounds against influenza virus. To dissect the relationship between the antiviral mechanism of action and the structure of our newly designed virucidal we compare, for the first time, its efficacy with that of a highly similar compound displaying a virustatic activity. We show that if the inhibitory effect is reversible, that is, virustatic, the antiviral efficacy is lost when moving from in vitro to ex vivo. On the other hand, the virucidal counterpart of the same molecule keeps its efficacy is maintained from in vitro all the way to in vivo. Importantly, we show in vivo results with remarkable effect on mice survival for a pandemic strain of H1N1 with the compound given intranasally 24h post‐infection. Therefore, we believe that our approach to design non‐toxic virucidal macromolecules has outstanding potential for the prevention and the treatment of not only human, but also avian influenza infections.

## Experimental Section

Detailed information on the experimental procedures can be found in the Supporting Information.

## Conflict of Interest

O.K., V.C., C.T., and F.S. are inventors on patent number EP18192559.5. All the other authors declare no conflict of interest.

## Author Contributions

O.K., V.C., C.T., and F.S. contributed equally to this work. Conceptualization, O.K., V.C., C.T., and F.S.; Methodology, O.K., V.C., and R.L.G. Investigation, O.K. and Y.Z. synthetized the cyclodextrins; O.K., V.C., J.B., J.M., and C.M. performed the in vitro experiments; V.C. performed the ex vivo experiments; L.S., B.D.C., and R.L.G. performed the in vivo experiments. Writing – Original Draft, O.K., V.C., C.T., and F.S. Writing – Review & Editing, all authors. Funding Acquisition, C.T., L.K., and F.S.; Resources, S.H. and S.C. Supervision, W.L.J.H., A.H., R.L.G., C.T., and F.S.

## Supporting information

Supporting Information
